# The Impact of Dietary Nutrient Intake on Red Blood Cell Distribution Width-Coefficient of Variation in Pregnant Women: A Cross-Sectional Observational Pilot Study

**DOI:** 10.3390/nu17213396

**Published:** 2025-10-29

**Authors:** Katarzyna Antosiak-Cyrak, Anna Demuth, Urszula Czerniak, Joanna Ratajczak, Ewa Bryl, Piotr Kowalski, Krystian Wochna, Magdalena Lewandowska, Katarzyna Domaszewska

**Affiliations:** 1 Department of Swimming and Water Lifesaving, Faculty of Sport Sciences, Poznan University of Physical Education, 61-871 Poznan, Poland; kwochna@awf.poznan.pl; 2 Department of Human Biological Development, Faculty of Sport Sciences, Poznan University of Physical Education, 61-871 Poznan, Poland; demuth@awf.poznan.pl (A.D.); czerniak@awf.poznan.pl (U.C.); jratajczak@awf.poznan.pl (J.R.); bryl@awf.poznan.pl (E.B.); 3 Department of Physiology and Biochemistry, Poznan University of Physical Education, 61-871 Poznan, Poland; kowal94p@gmail.com (P.K.); domaszewska@awf.poznan.pl (K.D.); 4 Calculation Centre, Poznan University of Physical Education, 61-871 Poznan, Poland; lewandowska@awf.poznan.pl

**Keywords:** nutrition, pregnancy, maternal diet, gestational nutrition, prenatal supplementation, pregnancy outcomes, RDW-CV, micronutrient intake

## Abstract

Background/Objectives: Dietary intake during pregnancy influences hematological parameters, reflecting nutritional status and potentially affecting maternal and fetal outcomes. This cross-sectional pilot study aimed to compare dietary nutrient intake in pregnant women depending on their red cell distribution width-coefficient of variation (RDW-CV). Methods: A total of 31 pregnant women in their third trimester were divided into the following two groups: within reference range RDW-CV (*n* = 22) and elevated RDW-CV (*n* = 9). Dietary intake was assessed via a dietary recall (USDA Multiple-Pass Method). Intake of energy (kcal/day), macronutrients (g/day), vitamins (mg/day; µg/day) B1, B3, B6, B9, B12, C, A, D, E, and minerals (mg/day) Zn, P, Mg, K, Ca, Fe were recorded. Complete blood count (CBC) parameters were measured (Total WBC, HGB, HCT, MCV, RDW-CV, PLT, NEU, LYM, MON, EOS, BAS, LMR). Results: The elevated RDW-CV group had a significantly lower level of daily energy adequacy. Both groups did not meet recommended intakes for energy, iron, vitamin D. A statistical significance in MCV differences was noted, with lower values in the elevated RDW-CV group, supported by a Cohen’s d = 0.82, suggesting early changes in erythrocyte size distribution. The reference range RDW-CV group consumed significantly more zinc, phosphorus, calcium, and vitamin B12, whereas vitamin C intake was higher in the elevated RDW-CV group. Conclusions: Pregnant women with an elevated RDW-CV tended to exhibit greater nutritional insufficiencies than those with reference range RDW-CV. Our findings suggest potential associations between hematologic indices (RDW-CV, MCV) and dietary nutrient intake patterns during pregnancy. These preliminary observations are based on a pilot study and warrant confirmation in larger, prospective studies incorporating biochemical markers.

## 1. Introduction

Anemia during pregnancy is among the most common health disorders affecting women of reproductive age and represents a major risk factor for adverse obstetric and perinatal outcomes. According to the World Health Organization (WHO), more than 36% of pregnant women worldwide suffer from some form of anemia, with iron deficiency anemia (IDA) being the predominant type [1,2,3]. The prevalence of anemia during pregnancy ranges from 17 to 31% in Europe and North America, 44 to 53% in Southeast Asia, and 53 to 61% in African [1,2,3].

Iron-deficiency-related anemia during pregnancy can lead to serious health consequences, including an increased risk of preterm delivery, low birth weight, and higher perinatal mortality [4,5]. Although iron deficiency accounts for approximately half of all anemia cases, the condition is often multifactorial and may coexist with deficiencies in other micronutrients [6]. In hematopoiesis, not only iron but also several other nutrients play essential roles, including vitamins A, B2, B6, B9 (folate), B12, C, D, and E, as well as trace elements such as copper [6,7,8,9,10,11]. Deficiencies in these nutrients may impair erythrocyte maturation, hemoglobin synthesis, and oxygen transport, thereby exacerbating existing hematological disturbances.

Previous research on anemia has primarily focused on micronutrient deficiencies; however, growing evidence indicates that macronutrients and overall energy balance also play a significant role in the regulation of erythropoiesis. Proteins, fats, and carbohydrates are crucial for metabolic regulation, mineral bioavailability, and the normal functioning of the hematopoietic system. An inadequate energy balance and improper macronutrient ratios may negatively influence pregnancy outcomes, fetal growth, and the long-term risk of chronic disease [4,12].

In the context of erythropoiesis, particular attention has been paid to hepcidin—a hormone regulating iron metabolism. Energy deficiency and imbalanced macronutrient intake have been shown to increase hepcidin concentrations, thereby reducing iron absorption and mobilization, and impairing red blood cell production [13,14,15]. Conversely, a well-balanced diet during pregnancy supports the physiological reduction in hepcidin levels, enhancing iron bioavailability and promoting normal erythropoiesis [16]. In contrast, unbalanced energy or macronutrients intake (e.g., low-carbohydrate or low-fat diets) may elevate hepcidin levels and lead to hematological disturbances, even in the presence of adequate iron intake [17]. Therefore, assessing energy balance and macronutrient composition may serve as a valuable complement to standard anemia diagnostics in pregnancy, providing important insights for nutritional interventions [18].

Laboratory indicators of iron deficiency anemia include the following: hemoglobin (Hb) concentration < 11 g/dL, serum ferritin < 30 ng/mL, decreased serum iron, increased transferrin concentration and total iron-binding capacity (TIBC), as well as decreased transferrin saturation (TSAT) and mean corpuscular volume (MCV) below 80 fL. [19]. Another useful parameter in the red cell distribution width (RDW-CV), whose reference values typically range between 11.5% and 14.5%. Elevated RDW-CV values are associated not only with various types of anemia [20,21,22] but also with poorer outcomes in several chronic diseases, including cardiovascular conditions. Recent evidence suggests that increased RDW-CV reflects subtle alterations in erythrocyte production and maturation that may occur before a decline in hemoglobin concentration, making it a potential early marker of disrupted hematopoiesis and nutrient deficiencies [23,24,25].

The mechanism underlying these changes involves alterations in erythrocyte morphology in response to nutrient deficiencies. Under normal conditions, the bone marrow produces red blood cells with a mean corpuscular volume (MCV) of approximately 90 ± 10 fL and a relatively low variability (RDW-CV ~12%). When iron deficiency develops, the bone marrow produces microcytes (MCV~60 ± 7 fL), which increases RDW-CV long before MCV, and hemoglobin values fall below the normal range. Similarly, folate or vitamin B12 deficiency results in macrocytosis, leading to elevated RDW-CV prior to the onset of overt morphological abnormalities [16,26,27].

Among pregnant women, studies assessing the clinical significance of elevated RDW-CV remain limited. Available data suggest that increased RDW-CV may be associated with deficiencies in both micro- and macronutrients that influence erythropoiesis and iron homeostasis. Such disturbances can impair red blood cell maturation and have been linked to an increased risk of preterm birth [28], intrauterine growth restriction (IUGR) [29,30], and other adverse perinatal outcomes. In fetuses and newborns, maternal nutritional deficiencies and disordered erythropoiesis may contribute to low birth weight, prematurity, neurocognitive impairment, and an elevated risk of neonatal anemia [26,31].

Given that RDW-CV is a simple, inexpensive, and routinely measured hematological parameter, it may serve as a valuable tool for the early identification of hematological and nutritional disturbances in pregnant women. Examining the association between nutrient intake and RDW-CV values could provide important insights for developing effective dietary interventions to support maternal hematological health and optimal fetal development.

A deeper understanding of the relationship between nutrient intake and RDW-CV could contribute to the formulation of targeted dietary recommendations aimed at optimizing the hematological status of pregnant women. Therefore, the aim of this pilot study was to compare nutrient intake from dietary sources among pregnant women according to their RDW-CV values. We hypothesized that women with elevated RDW-CV would demonstrate a lower dietary adequacy of key micro- and macronutrients compared with those with reference range RDW-CV values.

## 2. Materials and Methods

### 2.1. Characteristics of the Study Participants

This cross-sectional observational study was conducted between 2022 and 2023. Participants were recruited by hospital staff who enlisted a number of women admitted to a maternity ward in Sulęcin (Poland). Only women with healthy, low-risk pregnancies were eligible for participation. Exclusion criteria included the following: gestational age < 38 weeks, diabetes requiring special diet, multiple pregnancy, and adverse obstetric history. Additionally, only individuals who did not use dietary supplements during the third trimester were included. In total, 31 women in the third trimester of pregnancy (38–41 weeks of gestation) were enrolled in the study. Two women were excluded due to being under 18 years of age, and one woman was excluded because her reported caloric intake exceeded 3500 kcal per day, which was above the inclusion threshold. The sample size was determined based on the pilot and the feasibility of the study, which aimed to explore preliminary associations and assess the practicality of recruitment and data collection procedures for a larger future study. As complete blood count (CBC) testing was part of the standard medical procedures performed upon hospital admission, participants provided written informed consent for the use and analysis of their CBC results in combination with the dietary data collected during the study. All participants were fully informed about the purpose of linking these data sources and the scope of the research, ensuring voluntary participation and data confidentiality.

### 2.2. Complete Blood Count

Blood samples were collected after a 12 h overnight fast at 7:00 a.m., while participants were at rest, using S-Monovette syringes (Sarstedt, Nümbrecht, Germany). The following parameters were determined immediately after collection: hemoglobin (HGB) concentration, haematocrit (HCT), mean corpuscular volume (MCV), white blood cell count (WBC), red cell distribution width-coefficient of variation (RDW-CV), platelet count (PLT), and the relative proportion of neutrophils (NEU), lymphocytes (LYM), monocytes (MON), eosinophils (EOS), and basophils (BAS). Based on the obtained leukocyte differential counts, the lymphocyte-to-monocyte ratio (LMR) was calculated as an indicator of systemic inflammatory status and interpreted according to [20]. Analyses were performed using an automated hematology analyzer, “Sysmex XS-1000i” (Sysmex Corporation, Kobe, Japan).

### 2.3. Nutritional Analysis

Dietary intake was assessed using a dietary recall conducted according to the USDA Multiple-Pass Method, a validated tool for collecting short-term dietary intake data. Interviews were performed on the day of hospital admission by a trained dietitian to ensure accuracy and consistency. The collected data included mealtimes, types of consumed foods, beverages and snacks, preparation methods, and portion sizes expressed in grams or using household measurements. All recalls were collected on typical weekdays to minimize atypical consumption patterns. Participants confirmed that the recalled weekday represented their typical dietary intake and did not differ from their usual eating patterns. In cases of uncertainty, the interviewer returned to specific questions at the end of the session to verify the responses. Implausible energy intake values were identified and excluded using the commonly applied thresholds of <500 kcal or >3500 kcal per day [32]. Nutrient intake recommendations were based on the reference Estimated Average Requirement (EAR) values for the Polish population, as defined under the editorship of Ewa Rychlik and endorsed by the National Institute of Public Health [33]. The EAR represents an intake level sufficient for approximately 50% of individuals in a given group, allowing for the estimation of the proportion of individuals whose consumption falls below this threshold and thus are at potential risk of deficiency [34]. Nutrients densities were additionally expressed per 1000 kcal to standardize the intake relative to total energy consumption. The normalization was performed only for nutrients that showed significant differences between the study groups, enabling the differentiation between the effects related to overall energy intake and those reflecting actual nutrient density.

Dietary data were analyzed using the Nuvero web application (NUVERO-VOIX, Koszalin, Poland; accessed: May 2022), which is based on the database of the National Institute of Public Health–National Institute of Hygiene in Warsaw [35]. The analysis included the following: daily intake of energy (kcal/day); macronutrients (%E), namely carbohydrates, fats, and proteins; vitamins (mg/day; µg/day), namely B1, B3, B6, B9, B12, C, A, D, and E; and minerals (mg/day), namely zinc (Zn), phosphorus (P), magnesium (Mg), potassium (K), calcium (Ca), and iron (Fe), all of which were evaluated in relation to dietary guidelines.

### 2.4. Statistical Analysis

Calculations were performed using TIBCO Software Inc. (2017) Statistica (data analysis software system version 13, Tulsa, OK, USA. Descriptive statistics were expressed in terms of mean value and standard deviation (M ± SD). Additionally, 95% confidence intervals (95% CI) were reported to estimate the precision of the potential range of observed effects within the population. The normality of data distribution was verified using the Shapiro–Wilk test, and the homogeneity of variance was assessed using Levene’s test. Depending on the results, the differences between women with RDW-CV values within the reference range and those with elevated RDW-CV were evaluated using Student’s *t*-test, analysis of variance, or the nonparametric Mann–Whitney U test. The effect sizes (Cohen’s d) were calculated as the ratio of the mean difference to the standard deviation of the difference, and categorized according to Cohen’s criteria as small (≥0.2 and <0.5), moderate (≥0.5 and <0.8), or large (≥0.8) [36,37]. Cohen’s d was reported alongside *p*-values to provide an estimate of the magnitude of observed effects, independent of sample size. Statistical significance was set at *p* < 0.05.

## 3. Results

### 3.1. Group Characteristics

Participants were categorized into the following two groups based on their RDW-CV values: within the reference range (11.5–14.5%) and elevated (≥14.5%), following established reference cut-off values [21]. The reference range RDW-CV group included twenty-two women, with a mean RDW-CV of 13.43 ± 0.73%. The elevated RDW-CV group included nine women, with a mean RDW-CV of 15.43 ± 0.68%. Both groups were comparable in terms of age (*p* ≥ 0.05). The mean age in the reference range RDW-CV group was 30.09 ± 4.79 years, while in the elevated RDW-CV group it was 26.11 ± 5.42 (t = 2.02; *p* = 0.052). Similarly, gestational age did not differ significantly between groups (40.14 ± 1.21 vs. 40.33 ± 0.87 weeks; Z = −0.24; *p* = 0.810).

### 3.2. Complete Blood Count (CBC)

Table 1 presents the comparison of basic CBC parameters between pregnant women with reference range and elevated RDW-CV values. A statistically significant difference was observed in mean corpuscular volume (MCV, fL) between the two groups (*p* < 0.05), with higher values in the reference range RDW-CV group (∆MCV = 3.33 fL. The corresponding effect size indicated a large effect (Cohen’s d = 0.82). No statistically significant differences (*p* ≥ 0.05) or trends toward significance were found for other CBC parameters. Similarly, the inflammatory marker lymphocyte-to-monocyte ratio (LMR) did not differ between the groups (*p* ≥ 0.05).

### 3.3. Macronutrients

Table 2 presents a comparison of energy adequacy and macronutrient intake between pregnant women with RDW-CV values within the reference range and those with elevated RDW-CV. Women in the reference range RDW-CV group demonstrated significantly higher energy adequacy, with an intergroup difference of 378.52 kcal (Cohen’s d = 1.01), indicating a large effect size. In this group, the mean protein intake accounted for 17.35% of the total energy intake, whereas in the elevated RDW-CV group it was slightly lower (16.79%), though still within the recommended range. The mean carbohydrate intake was 51.92% in the reference range RDW-CV group and 55.05% in the elevated RDW-CV group, while the mean total fat intake was 31.73% and 28.16%, respectively. No statistically significant differences (*p* ≥ 0.05) in macronutrient intake were observed between the two groups.

### 3.4. Micronutrients

The analysis of selected micronutrients and vitamin intake revealed significant differences between the two RDW-CV groups (Table 3). The intake of the following micronutrients was significantly higher in the reference range RDW-CV group: zinc (mg/day), with an intergroup difference of 1.87 mg/day (Cohen’s d = 0.88); phosphorus (mg/day), with a difference of 325.54 mg/day (Cohen’s d = 1.39); calcium (mg/day), with a difference of 206.43 mg/day (Cohen’s d = 1.34); and vitamin B12 (µg/day), with a difference of 1.17 µg/day (Cohen’s d = 2.31). The only exception was vitamin C (mg/day), for which intake was significantly lower in the reference range RDW-CV group, with a difference of 72.38 mg/day (Cohen’s d = 2.32). For all parameters demonstrating statistically significant differences (*p* < 0.05), the values of Cohen’s d exceeded 0.8, indicating a very large effect size between the groups.

To account for the differences in total energy intakes, nutrient consumption was normalized per 1000 kcal of diet (Table 4.). In the elevated RDW-CV group, a tendency toward a lower intake of phosphorus, calcium, and vitamin B12 was observed; however, these differences did not reach statistical significance. After adjusting for nutrient density per 1000 kcal, only vitamin C intake remained at a significantly higher intake level in the elevated RDW-CV group. The difference in vitamin C intake was 56.28 mg per 1000 kcal, and the corresponding Cohen’s d value (d = 1.35) indicated a strong intergroup effect.

**Figure 1:**
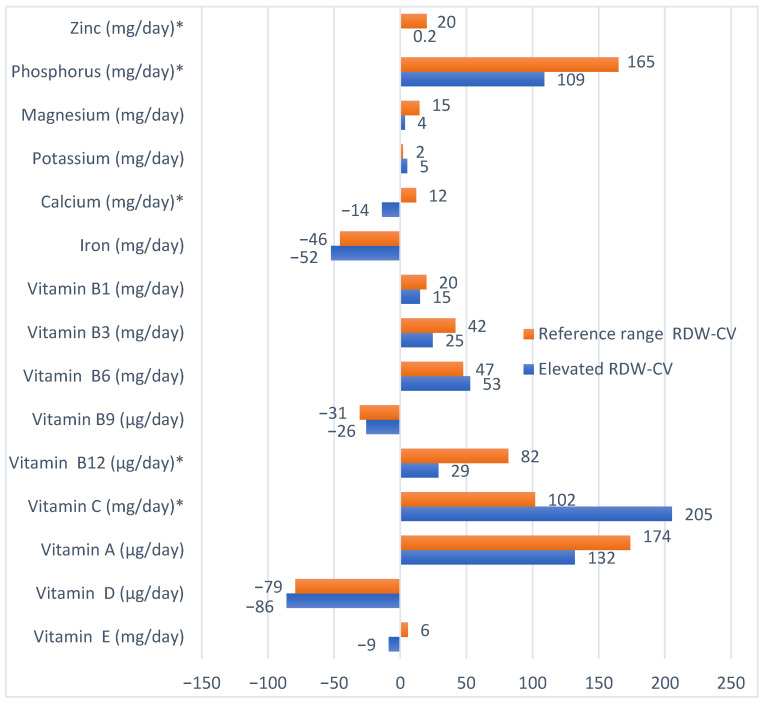
Implementation of EAR standards by RDW-CV category. Note: * *p* < 0.05—statistically significant differences.

The analysis of the compliance with the EAR standards according to RDW-CV categories (Figure 1) revealed that most dietary micronutrients were consumed in amounts within the recommended range. However, calcium, iron, folate, and vitamin D intake remained below the reference values, with vitamin D intake in the elevated RDW-CV group reaching −86% of the EAR. In contrast, vitamin C intake in this group amounted to 205% of the reference values.

## 4. Discussion

The only statistically significant difference in the hematological parameters between the groups within reference range and with elevated RDW-CV was the mean corpuscular volume (MCV), which was lower in the elevated RDW-CV group (87.20 ± 3.34 fL vs. 90.53 ± 4.28 fL; *p* = 0.046). Although these values remained within the physiological range for pregnancy (81–99 fL), the reduced MCV may indicate early microcytic changes, often associated with iron deficiency—one of the most common nutrient deficiencies among pregnant women. However, it should be emphasized that this study was a pilot investigation conducted on a relatively small, cross-sectional sample; therefore, the results should be interpreted as preliminary and indicative of directions for further research. Hemoglobin (HGB) and hematocrit (HCT) levels, although not significantly different between groups (*p* = 0.195 and *p* = 0.618, respectively), were below the recommended reference ranges for pregnancy. In particular, in the elevated RDW-CV group, the mean HGB was 10.44 ± 0.83 g/dL, falling below the lower limit of normal (11.0 g/dL) [38]. Similarly, HCT values in both groups (32.17 ± 2.99% and 30.62 ± 3.40%) were slightly below the accepted physiological range for pregnancy (33–43%) [39]. According to the literature, HCT levels between 30 and 32% [40] may reflect physiological hemodilution and should prompt monitoring of ferritin and iron levels, whereas a decrease below 30% warrants further diagnostics for iron deficiency anemia (IDA) and consideration of iron supplementation) [41]. Other hematological parameters—including white blood cell count (WBC), platelet count (PLT), and differential counts (neutrophils, lymphocytes, monocytes, eosinophils, and basophils)—remained within the established reference ranges for pregnancy and did not differ significantly between the analyzed groups (all *p* > 0.05). These findings are consistent with typical hematological adaptations in pregnancy, such as physiological leukocytosis and neutrophilia.

In this study, pregnant women with elevated RDW-CV were characterized by a lower dietary energy intake and a reduced consumption of several key micronutrients (zinc, calcium, phosphorus, and vitamin B12) compared to women who were within reference range RDW-CV. These findings suggest a potential relationship between diet quality and red blood cell volume variability, reflecting both nutritional status and overall metabolic condition.

The results indicate that women with elevated RDW-CV did not meet their estimated energy requirements by approximately 400 kcal/day. Although this difference was only moderately significant statistically, it may hold clinical relevance, as chronic energy deficiency during pregnancy can lead to adaptive changes in hematopoiesis and disturbances in erythropoiesis [18,42]. Inadequate intake of energy and protein may also impair the utilization of micronutrients such as iron and zinc, thereby increasing red blood cell size variability and contributing to elevated RDW-CV.

The most consistent pattern observed in our study was the coexistence of lower energy intake alongside deficiencies in selected minerals (calcium, zinc, phosphorus) and vitamin B12. This dietary profile corresponds to the so-called “suboptimal Western diet,” characterized by a high proportion of processed foods and the insufficient consumption of fresh protein sources and dairy products. Previous studies [43,44,45] have indicated that low calcium and zinc levels in pregnant women may independently contribute to the deterioration of red blood cell indices, including elevated RDW-CV.

RDW-CV variability may reflect not only micronutrient deficiencies but also inflammatory processes and oxidative stress. Increases in RDW have been observed in conditions associated with the elevated production of proinflammatory cytokines (IL-6, TNF-α), which impair iron absorption and inhibit red blood cell maturation [46]. Oxidative stress—heightened in late pregnancy—may further accelerate red blood cell degradation and increase volume heterogeneity [47]. Additionally, iron redistribution toward immune tissues (a host defense mechanism) leads to functional iron deficiency in the bone marrow, thereby increasing RDW independently of dietary factors [48].

In the present analysis, inflammatory markers—specifically LMR—did not differ significantly between the groups (*p* ≥ 0.05), suggesting that the observed RDW-CV changes were largely independent of active inflammation. Since the LMR values were similar in both groups and within the normal range, it can be assumed that the observed differences in RDW-CV were not due to the presence of inflammatory processes. Future studies would benefit from the concurrent measurement of additional inflammatory biomarkers (e.g., CRP, hepcidin) and oxidative stress indicators to further verify these hypotheses.

Our findings are consistent with those reported by Szamotulska and Mierzejewska [43] and Iglesias-Vázquez et al. [45], who also demonstrated the insufficient intake of calcium, vitamin D, and iron among pregnant women in Poland and Spain. Differences in vitamin C and B12 intake may reflect cultural and seasonal dietary variations. Aparicio et al. [49] similarly reported an association between low vitamin B12 intake and elevated RDW, confirming the crucial role of this vitamin in maintaining normal erythropoiesis.

The observed differences in energy intake (approximately 380 kcal/day) and selected nutrients (e.g., calcium by 206.43 mg/day and zinc by 1.87 mg/day) may have important clinical implications. Deficiencies of this magnitude may disrupt the energy–mineral balance, predisposing to iron and other micronutrient deficiency anemias. Elevated RDW-CV in these women may, therefore, serve as an early indicator of subclinical deficiencies, even before overt anemia develops. This supports the potential value of RDW as a simple and accessible biomarker for assessing nutritional quality during pregnancy.

Further research studies with larger sample sizes and greater statistical power are warranted. Such studies should include inflammatory biomarkers, oxidative stress indicators, and serum vitamin and mineral concentrations. In our analysis—which involved standardizing nutrient intake per 1000 kcal to account for difference in total energy intake—trends toward a lower intake of phosphorus, calcium, and vitamin B12 were observed in the elevated RDW-CV group, although these differences were not statistically significant. Only vitamin C intake was significantly higher in this group (difference: 56.28 mg per 1000 kcal), with a high Cohen’s d effect size (d = 1.35) indicating a strong between-group effect. Further studies combining dietary data with biomarkers of inflammation and oxidative stress could clarify how maternal nutrition affects red blood cell variability and metabolic factors in pregnancy. No formal correction for multiple comparisons was applied due to the exploratory, pilot nature of the study. We acknowledge that this increases the risk of Type I error, and these findings should therefore be interpreted with caution. Although some comparisons did not reach statistical significance, several effect sizes indicated moderate to large differences between groups. This pattern may reflect insufficient statistical power rather than lack of biological relevance. Future research with larger sample sizes should apply multivariate regression and corrections for multiple testing to confirm the robustness of the observed associations.

A diet characterized by lower energy adequacy and the insufficient intake of calcium, zinc, and vitamin B12 is associated with elevated RDW-CV in pregnant women. These findings underscore the importance of both quantitative and qualitative aspects of nutrition in maintaining optimal hematological parameters during pregnancy. Nevertheless, it should be acknowledged that alterations in RDW may also arise from non-dietary factors, such as inflammation or oxidative stress. Integrating comprehensive nutritional assessment with hematological and inflammatory biomarkers may therefore represent a valuable strategy for identifying women at increased risk of nutrient deficiencies and disturbances in erythropoiesis during pregnancy. We suggest that, if confirmed by larger studies incorporating biochemical validation, RDW-CV could complement existing screening tools for identifying women at risk of suboptimal nutrition.

### Limitations

This pilot study has several notable strengths. Its integrative approach combined a comprehensive assessment of complete blood count (CBC) parameters with detailed macro- and micronutrient intake data, allowing for more nuanced understanding of potential relationships between diet and hematological status in pregnant women. Stratification based on RDW-CV reference values facilitated the clearer identification of differences in both the hematological and dietary parameters, enhancing the interpretability of the findings. The study also examined specific nutrients, including key vitamins and minerals, providing insight into dietary influences on hematological outcomes. The inclusion of effect size analysis (Cohen’s d) further strengthened the interpretative value beyond *p*-values, highlighting clinically relevant trends despite the small sample size.

Several limitations should be acknowledged. The cross-sectional design precludes causal inferences between nutrient intake and hematological outcomes. We did not collect supplementary variables, such as gestational age, BMI, and parity, which could allow preliminary evaluation of potential confounding factors. The elevated RDW-CV group was small (*n* = 9), which reduced statistical power and increased the risk of Type I and Type II errors. Nutrient intake was self-reported, potentially introducing recall bias and under- or overestimation of actual consumption. Single-day dietary recalls do not fully capture habitual intake; nevertheless, they provide valuable insight into current dietary practices, particularly in relatively homogeneous populations at specific physiological stages, such as late pregnancy. They also enable the assessment of diet quality and the adherence to nutritional recommendations. However, the recruitment during childbirth admission may have influenced dietary reporting due to stress, potentially introducing additional recall bias. Participants reporting dietary supplement use were excluded, and no structured questionnaire or biochemical verification (e.g., ferritin, vitamin D, and vitamin B12) were conducted, which limits the ability to confirm micronutrient status. The exclusion of participants using dietary supplements may also limit the generalizability of our findings, as supplementation is common in prenatal care. Finally, the absence of sociodemographic data, such as education, socioeconomic status, and lifestyle factors, restricts the capacity to control for potential confounders.

Despite these limitations, the study provides valuable preliminary data on diet–hematology relationships in late pregnancy and can inform the design of future larger-scale studies incorporating longitudinal assessment, biochemical markers, and broader demographic variables to enhance both the interpretability and generalizability of findings.

## 5. Conclusions

Pregnant women with an elevated RDW-CV tended to exhibit greater nutritional insufficiencies than to those with reference range RDW-CV. Our findings suggest potential associations between hematologic indices (RDW-CV, MCV) and dietary nutrient intake patterns during pregnancy. These preliminary observations are based on a pilot study and warrant confirmation in larger, prospective studies incorporating biochemical markers such as CRP, ferritin, vitamin D, and folate.

## Figures and Tables

**Table 1:** A comparison of basic CBC parameters by RDW-CV category.

Parameters	RIs *	RDW-CV		
		Reference Range	Elevated	*p*-Value
Total WBC (10^3^/µL)	5.7–15	11.70 ± 3.51 (10.14; 13.25)	12.51 ± 3.55 (9.78; 15.24)	0.327 ^b^
HGB (g/dL)	11–14	11.02 ± 1.18 (10.5; 11.54)	10.44 ± 0.83 (9.8; 11.08)	0.195 ^a^
HCT (%)	28–40	32.17 ± 2.99 (30.84; 33.49)	31.60 ± 2.43 (29.73; 33.47)	0.618 ^a^
MCV (fL)	82–92	90.53 ± 4.28 (88.64; 92.43)	87.2 ± 3.34 (84.63; 89.77)	**0.046 ^a^**
PLT (10^3^/mm^3^)	150–400	223.59 ± 69.42 (192.81; 254.37)	234 ± 48.58 (196.66; 271.34)	0.686 ^a^
NEU (%)	40–80	72.58 ± 4.43 (70.62; 74.54)	73.24 ± 6.97 (67.89; 78.6)	0.752 ^a^
LYM (%)	20–45	18.39 ± 4.65 (16.33; 20.45)	17.41 ± 5.61 (13.1; 21.73)	0.619 ^a^
MON (%)	2–8	8.02 ± 1.65 (7.28; 8.75)	8.16 ± 1.54 (6.97; 9.34)	0.832 ^a^
EOS (%)	2–4	1.07 ± 0.85 (0.69; 1.44)	1.09 ± 0.96 (0.35; 1.83)	0.794 ^b^
BAS (%)	0.5–1	0.21 ± 0.11 (0.16; 0.26)	0.18 ± 0.07 (0.13; 0.23)	0.586 ^b^
LMR	2–10	2.30 ± 0.74 (2.05; 2.79)	2.16 ± 0.75 (1.58; 2.74)	0.462 ^a^

Data are presented as M ± SD; (95% CI); ^a^ Student’s *t*-test; ^b^ Mann–Whitney U test; *p* < 0.05—statistically significant differences are highlighted in bold; RDW-CV—Red Cell Distribution Width-Coefficient of Variation; WBC—White Blood Cell; HGB—Hemoglobin; HCT—Haematocrit; MCV—Mean Corpuscular Volume; PLT—Platelet Count; NEU—Neutrophils; LYM—Lymphocytes; MON—Monocytes; EOS—Eosinophils; BAS—Basophils; LMR—Lymphocytes-to-Monocyte ratio; and * Ris—Reference intervals.

**Table 2:** A comparison of energy adequacy by RDW-CV category.

Parameters	% E *	RDW-CV		*p*-Value
		Reference Range	Elevated	
Energy (Kcal)	-	2159.41 ± 431.24 (1968.21; 2350.61)	1780.89 ± 306.55 (1545.26; 2016.52)	**0.039 ^b^**
Proteins (%)	10–20	17.35 ± 2.66 (16.17; 18.53)	16.79 ± 1.81 (15.40; 18.18)	0.571 ^a^
Fats (%)	20–35	30.73 ± 4.80 (28.60; 32.86)	28.16 ± 7.37 (22.49; 33.83)	0.257 ^a^
Carbohydrates (%)	45–65	51.92 ± 5.53 (49.47; 54.37)	55.05 ± 6.67 (49.92; 60.17)	0.188 ^a^

Data are presented as M ± SD; (95% CI); ^a^ Student’s *t*-test; ^b^ Mann–Whitney U test; *p* < 0.05—statistically significant differences are highlighted in bold; RDW-CV—Red Cell Distribution Width-Coefficient of Variation; and * % E Percentage of Energy.

**Table 3:** A comparison of selected micronutrient and vitamin intake by RDW-CV category.

Parameters	EAR/AI *	RDW-CV			*p*-Value
		Reference Range	↑ **	Elevated	
Zinc (mg/day)	9.5	11.39 ± 2.27 (10.38; 12.39)		9.52 ± 1.71 (8.20; 10.84)	**0.035 ^a^**
Phosphorus (mg/day)	580	1536.48 ± 247.24 (1426.86; 1646.1)		1210.94 ± 198.30 (1058.52; 1363.37)	**0.002 ^a^**
Magnesium (mg/day)	300	343.50 ± 86.34 (305.22; 381.78)		310.67 ± 75.01 (253.01; 368.32)	0.305 ^c^
Potassium (mg/day)	3500 *	3572.80 ± 694.50 (3264.88; 3880.72)		3680.38 ± 670.80 (3164.76; 4196)	0.983 ^b^
Calcium (mg/day)	800	895.76 ± 155.25 (826.93; 964.6)		689.33 ± 150.93 (573.32; 805.35)	**0.002 ^a^**
Iron (mg/day)	23	12.52 ± 3.20 (11.10; 13.93)		10.94 ± 2.45 (9.06; 12.82)	0.196 ^a^
Vitamin B1 (mg/day)	1.2	1.44 ± 0.46 (1.23; 1.64)		1.38 ± 0.32 (1.14; 1.62)	0.983 ^b^
Vitamin B3 (mg/day)	14	19.84 ± 5.45 (17.42; 22.25)		17.43 ± 3.31 (14.89; 19.98)	0.230 ^a^
Vitamin B6 (mg/day)	1.6	2.36 ± 0.53 (2.13; 2.59)		2.44 ± 0.44 (2.10; 2.78)	0.673 ^a^
Vitamin B9 (µg/day)	520	360.16 ± 90.87 (319.87; 400.45)		386.48 ± 106.96 (304.26; 468.7)	0.492 ^a^
Vitamin B12 (µg/day)	2.2	4.00 ± 1.46 (3.35; 4.64)		2.83 ± 0.83 (2.20; 3.47)	**0.024 ^b^**
Vitamin C (mg/day)	70	141.35 ± 87.21 (102.68; 180.02)	↑	213.73 ± 74.57 (156.41; 271.05)	**0.039 ^b^**
Vitamin A (µg/day)	530	1451.16 ± 573.53 (1196.87; 1705.45)		1229.08 ± 418.35 (907.51; 1550.65)	0.303 ^a^
Vitamin D (µg/day)	15 *	3.08 ± 2.00 (2.19; 3.97)		2.10 ± 0.74 (1.53; 2.67)	0.184 ^b^
Vitamin E (mg/day)	10 *	10.57 ± 3.82 (8.88; 12.26)		9.12 ± 2.77 (6.99; 11.25)	0.313 ^a^

Data are presented as M ± SD; (95% CI); ^a^ Student’s *t*-test; ^b^ Mann–Whitney U test; ^c^ variance estimation test; *p* < 0.05—statistically significant differences are highlighted in bold; RDW-CV—Red Cell Distribution Width-Coefficient of Variation; EAR—Estimated Average Requirement; * AI—Adequate Intake; and ** ↑ higher in the elevated RDW-CV group.

**Table 4:** A comparison of selected micronutrient density and vitamin intake by RDW-CV category.

Parameters	RDW-CV			*p*-Value
	Reference Range	↑ *	Elevated	
Zinc (mg/1000)	5.33 ± 0.85 (4.95; 5.71)		5.44 ± 1.02 (4.65; 6.22)	0.766 ^a^
Phosphorus (mg/1000)	725.71 ± 128.28 (668.84; 782.59)		691.75 ± 122.17 (597.84; 785.66)	0.503 ^a^
Calcium (mg/1000)	425.93 ± 85.78 (387.89; 463.96)		398.24 ± 106.41 (316.45; 480.03)	0.453 ^a^
Vitamin B12 (µg/1000)	1.92 ± 0.88 (1.53; 2.31)		1.60 ± 0.46 (1.25; 1.96)	0.396 ^b^
Vitamin C (mg/1000)	65.58 ± 40.07 (47.81; 83.35)	↑	121.86 ± 45.53 (86.86; 156.86)	**0.004 ^b^**

Data are presented as M ± SD; (95% CI); ^a^ Student’s *t*-test; ^b^ Mann–Whitney U test; *p* < 0.05—statistically significant differences are highlighted in bold; RDW-CV—Red Cell Distribution Width-Coefficient of Variation; and * ↑ higher in the elevated RDW-CV group.

## Data Availability

The data presented in this study is available on request from the corresponding author.

## Abbreviations

The following abbreviations are used in this manuscript:

CBC	Complete Blood Count
RIs	Reference Intervals
RDW-CV	Red Cell Distribution Width—Coefficient of Variation
WBC	White Blood Cell
TIBC	Total Iron-Binding Capacity
TSAT	Transferrin Saturation
HGB	Hemoglobin
HCT	Haematocrit
MCV	Mean Corpuscular Volume
PLT	Platelet Count
NEU	Neutrophils
LYM	Lymphocytes
MON	Monocytes
EOS	Eosinophils
BAS	Basophils
EAR	Estimated Average Requirement
EER	Estimated Energy Requirement
UI	Tolerable Upper Intake Level
AI	Adeqate Intake
Zinc	Zn
Phosphorus	P
Magnesium	Mg
Potassium	K
Calcium	Ca
Iron	Fe
Folic acid	B9
Riboflavin	B2
